# Mesenchymal stem cell interaction with Ti_6_Al_4_V alloy pre-exposed to simulated body fluid†

**DOI:** 10.1039/c9ra08912h

**Published:** 2020-02-13

**Authors:** Petra Jarolimova, Barbora Voltrova, Veronika Blahnova, Vera Sovkova, Eva Pruchova, Vojtech Hybasek, Jaroslav Fojt, Eva Filova

**Affiliations:** Department of Metals and Corrosion Engineering, Faculty of Chemical Technology, University of Chemistry and Technology Technická 5 166 28 Prague Czech Republic jarolime@vscht.cz; Department of Tissue Engineering, Institute of Experimental Medicine of the Czech Academy of Sciences Vídeňská 1083 Prague 4 142 20 Czech Republic; Faculty of Science, Charles University in Prague Albertov 2038/6 128 00 Prague Czech Republic; Second Faculty of Medicine, Charles University in Prague V Úvalu 84 150 06 Prague Czech Republic; University Centre for Energy Efficient Buildings, Czech Technical University in Prague Třinecká 1024 273 43 Buštěhrad Czech Republic

## Abstract

Titanium and its alloys are widely used for substitution of hard tissues, especially in orthopaedic and dental surgery. Despite the benefit of the use of titanium for such applications, there are still questions which must be sorted out. Surface properties are crucial for cell adhesion, proliferation and differentiation. Mainly, micro/nanostructured surfaces positively influence osteogenic differentiation of human mesenchymal stem cells. Ti_6_Al_4_V is a biocompatible α + β alloy which is widely used in orthopaedics. The aim of this study was to investigate the interaction of the nanostructured and ground Ti_6_Al_4_V titanium alloys with simulated body fluid complemented by the defined precipitation of hydroxyapatite-like coating and to study the cytotoxicity and differentiation capacity of cells with such a modified titanium alloy. Nanostructures were fabricated using electrochemical oxidation. Human mesenchymal stem cells (hMSC) were used to evaluate cell adhesion, metabolic activity and proliferation on the specimens. The differentiation potential of the samples was investigated using PCR and specific staining of osteogenic markers collagen type I and osteocalcin. Our results demonstrate that both pure Ti_6_Al_4_V, nanostructured samples, and hydroxyapatite-like coating supported hMSC growth and metabolic activity. Nanostructured samples improved collagen type I synthesis after 14 days, while both nanostructured and hydroxyapatite-like coated samples enhanced collagen synthesis on day 21. Osteocalcin synthesis was the most enhanced by hydroxyapatite-like coating on the nanostructured surfaces. Our results indicate that hydroxyapatite-like coating is a useful tool guiding hMSC osteogenic differentiation.

## Abstract 2

Titanium and its alloys are widely used for substitution of hard tissues, especially in orthopaedic and dental surgery.

## Graphical abstract

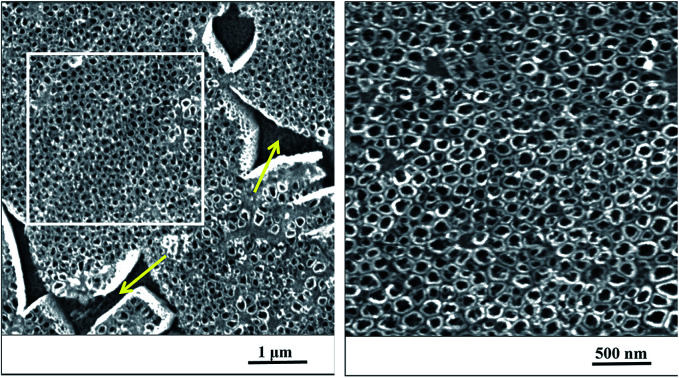

## Introduction

Titanium alloys are among the most advanced metallic biomaterials, used mainly in orthopaedics and dental implantology. Their greatest advantages are low density, high fatigue strength, biocompatibility and excellent resistance to corrosion.^1–3^ Several surface modifications, *e.g.* organic coatings, electrochemical deposition or plasma spraying of hydroxyapatite, *etc.* are used to enhance bioactivity, which is the result of the interaction of the material with the environment of the body, where a specific biological activity occurs.^4–6^ The mechanism of bioactivity consists of the formation of a layer which, by its composition and structure, approaches a bone hydroxyapatite (Ca_10_(PO_4_)_6_(OH)_2_) (HAp) and where the differentiation and proliferation of osteoblasts occurs.^7,8^

Another strategy for improving osseointegration is the preparation of nanostructured surface on titanium alloys. Electrochemical oxidation in fluorides containing electrolytes leads to self-organised TiO_2_ nanotube layer formation on the surface. It is possible to easily control the diameter and length of the nanotubes by adjusting experimental conditions, such as the electrolyte composition, potential setting or temperature. Diameters of nanotubes ranging between 20–100 nm are the most common.^9–12^ Selecting the most appropriate nanotube diameter is very important especially for cell interaction. Nanotubes of smaller diameters (15–30 nm) are known to support cell adhesion, while nanotubes of larger diameters (80–100 nm) have been reported to support osteogenic differentiation but can also result in programmed cell death.^13,14^ So changed nanotopography of the material can improve cell adhesion which is crucial for an implants osseointegration.^15^ The process of osseointegration is driven by supporting the adsorption of proteins such as vitronectin or fibronectin, which mediate the signals between cells and material surface. It is well known that cell adhesion is not influenced only by surface properties, but also by material chemistry and charge distribution.^16–18^

As mentioned above, the binding of bone and metal implants is mediated by the formation of an apatite layer on the implants surface. Therefore, the presence and morphology of the apatite layer can dramatically influence cell adhesion, differentiation and finally implant integration. Hydroxyapatite coated titanium showed better osteoinductivity and osteoconductivity as compared to untreated titanium surfaces.^19^ For successful implant integration, biologically effective ions Ca and P are very important for cell adhesion, differentiation and growth of osteogenic cells, and contribute to bone resorption and osteoclastogenesis as well.^20–23^

Various techniques for the coating of titanium and its alloys with Ca–P layer have been studied, including the sol–gel method,^24^ the ion beam assisted vapour deposition method,^25^ the plasma spraying method,^26^ the electrochemical deposition method^27^ and phase-transited lysozyme coating.^28^ The exposition of titanium in a simulated body fluid (SBF) in order to create Ca and P-containing layer was also investigated. It was revealed, that the presence of the nanostructure enhances calcium and phosphate deposition and titanium surfaces can be spontaneously covered with hydroxyapatite-like layer *via* exposure to SBF.^29^

The purpose of the present work was to study the interaction of human mesenchymal stem cells (hMSC) with HAp-like coating on ground and nanostructured surfaces of the Ti_6_Al_4_V alloy. To investigate the biocompatibility effect of nanostructured samples with and without the HAp-like coating, *in vitro* cultivation using human mesenchymal stem cells (hMSC) was applied, whereas a glass coverslip was used as reference.

## Experimental

### Samples preparation

Disc specimens of a Ti_6_Al_4_V alloy with a 16 mm diameter and a 3 mm height were ground using 2500 SiC paper and cleaned with ethanol and demineralised water in an ultrasonic bath. Nanotube preparation was managed by Galvanostat Jaissle IMP 88 PC-200V with the control unit PGU-AUTO Extern. A silver/silver chloride reference electrode with 3 mol L^−1^ KCl (ACLE) and two graphite rods as counter electrodes were used. The procedure consisted of a potential sweep of 100 mV s^−1^ to a 30 V potential, followed by a 2200 s delay at this potential. An ammonium sulphate (1 mol L^−1^) and ammonium fluoride (0.2 wt%) solution was used as the electrolyte. Samples were cleaned again after the exposition (sonication in demineralised water, ethanol and acetone). All anodization experiments were carried out at room temperature. For the morphological characterization of samples, a TESCAN VEGA 3 LMU scanning electron microscope (SEM) was used. The length and the inner diameter of the nanotubes were evaluated from the SEM images using ImageJ software. Data from four distant image fields from at least three samples were used for analysis.

### Exposure to simulated body fluid

Specimens with ground and nanostructured surface were exposed to simulated body fluid (Table 1), prepared according to Müller.^30^ Exposures were realized in 90 mL of SBF at a temperature of 37 °C for 168 hours. Experiments were supplemented by electrochemical measurements based on the defined sequence of electrochemical measurements. Measurements were performed using potentiostat Gamry Reference 600 with the Framework 6.25 control program. First, the hourly stabilization of the open circuit potential (OCP) was carried out, followed by repeated impedance monitoring at a constant frequency of 2 kHz and a polarization resistance measurement within ±20 mV/OCP. Once every 24 hours, impedance spectra were scanned at the frequency range 50 kHz to 10 mHz. After exposures, the samples were cleaned again with distilled water in ultrasonic bath. The pH value of SBF was recorded before and after each measurement. Subsequently, the specimens were observed using a TESCAN VEGA 3 LMU scanning electron microscope with an Oxford Instruments EDS analyzer. The results of the elemental analysis were evaluated using the Aztec program. After exposures to simulated body fluid, four sets of specimens were obtained and used for cell tests: ground Ti_6_Al_4_V (Ti), nanostructured Ti_6_Al_4_V (N), Ti_6_Al_4_V incubated in SBF (TiH), nanostructured Ti_6_Al_4_V incubated in SBF (NH). Glass was used as a reference group.

**Table 1:** Ion concentration in simulated body fluid (SBF)

Ion	Concentration [mmol dm^−3^]	Ion	Concentration [mmol dm^−3^]
Na^+^	142.0	HCO_3_^−^	27.0
K^+^	5.0	SO_4_^2−^	1.0
Ca^2+^	2.5	Cl^−^	109.0
Mg^2+^	1.5	HPO_4_^2−^	1.0

### Samples sterilization

After the exposure to SBF, the specimens were transferred to a 50 mL volumetric flask filled with a physiological saline solution. The samples were sterilized using a cyclic electron accelerator with Kapitza's resonator Microtron MT25 (Department of Accelerators-Nuclear Physics Institute of the Czech Academy of Sciences). An electron beam of 9.8 meV with a dose of 27 kGy was used.

### Cell seeding and cultivation

Human mesenchymal stem cells (hMSC, ScienceCell, Carlsbad, CA) were cultivated in basic medium consisting of: MEM Alpha medium (1X) (Gibco, Thermofisher Scientific, Waltham, MA, USA) supplemented with 10% (v/v) fetal bovine serum (FBS, Gibco), penicillin/streptomycin (100 IU mL^−1^ and 100 μg mL^−1^, Sigma-Aldrich) in CO_2_ incubator with 5% CO_2_ at the temperature of 37 °C.

After reaching 70% of confluence, the hMSC passage 4 were seeded on Ti_6_Al_4_V disc scaffolds and control glass coverslips at a density of 12 000 cells per cm^2^. The cells were cultured in basic medium. After 3 days of cell culture when the cells reached confluence, cultivating media were enriched by osteogenic differentiation additives: 40 μg mL^−1^ ascorbate-2-phosphate, 100 nM dexamethasone, and 10 mM glycerol-2-phosphate disodium salt. The osteogenic medium was changed twice a week.

### Cell metabolic activity measurement

The metabolic activity of the hMSC was determined on days 1, 7, 14 and 21 using the MTS assay (CellTiter96® AQueous One Solution Assay; Promega, Madison, WI, USA). Scaffolds were reloaded into new 12-well plate to avoid the distortion of results by cells occurring outside the scaffolds. To each scaffold, 800 μL of MTS solution in fresh medium were added. After incubation (37 °C, 5% CO_2_, 2 hours) the absorbance of the metabolised solution (100 μL) was detected at 490 nm using Tecan Infinite® PRO 200 series reader (Tecan, Männedorf, SUI). The background absorbance (690 nm) was subtracted from the measured data, as well as the absorbance of media with MTS solution.

### Immunohistochemical staining of type I collagen and osteocalcin

On day 14, and 21, the samples were fixed with frozen 70% methanol, washed with phosphate buffered saline (PBS) and incubated in 3% FBS in PBS/0.1% Triton-X for 30 min at RT. Primary monoclonal antibody against type I collagen (dilution 1 : 20, M-38-c, Developmental Studies Hybridoma bank, University of Iowa, Iowa City, Iowa, USA) or osteocalcin (dilution 1 : 20, osteocalcin 1–49 (human) IgG, T4743, Peninsula Laboratories, USA) was applied overnight at 2–8 °C. After 3 washes with PBS/0.05% Tween, and once in PBS, the samples were incubated with secondary antibody Alexa Fluor 488-conjugated anti-mouse antibody for 45 min, RT (4408S, Cell Signalling Technologies, dilution 1 : 400) or Alexa Fluor 633-conjugated anti-rabbit antibody (dilution 1 : 400, Life Technologies, USA). After washing three times with PBS/0.05% Tween, and once in PBS, cell nuclei were stained for 5 minutes with propidium iodide (RT, 5 μg mL^−1^ in PBS, Sigma Aldrich, USA) or using Hoechst 34580 (30 min at RT, dilution 1 : 5000, H21486, Life Technologies). After two washes with PBS, the cells were visualized using a confocal microscope ZEISS LSM 5 DUO at *λ*_exc_ = 488 nm and *λ*_em_ = 505–550 nm for Alexa Fluor 488, *λ*_ex_ = 405 nm, *λ*_em_ = 420–460 nm for Hoechst 34580, *λ*_exc_ = 633 nm and *λ*_em_ > 650 nm for Alexa Fluor 633.

Fluorescence intensity was counted from 8 to 9 photomicrographs from each sample group using MATLAB. The intensity was normalized for the number of cells and related to the mean of the control glass.

### Immunohistochemical staining of adhesive proteins vinculin and talin

hMSC were seeded as described in cell seeding. Two days after seeding, the cells were fixed with 4% paraformaldehyde/PBS for 5 min, washed once with PBS and stored in PBS at 2–8 °C before staining. The samples were then washed 3 times with PBS and incubated in 3% FBS/0.1% Triton X in PBS for 30 min at RT. The samples were then incubated with 1% Tween 20 in PBS for 20 min at RT. Monoclonal antibody against talin (dilution 1 : 200, clone 8d4, ascites fluid, T3287, Sigma-Aldrich) or vinculin (dilution 1 : 100, monoclonal hVIN-1, product V9131, Sigma-Aldrich) was applied overnight at 2–8 °C. After washing 3 times with PBS/0.05% Tween (3, 5, 10 min) and once in PBS, anti-mouse secondary antibody conjugated with goat anti-mouse IgG/IgA/IgM (H + L) Alexa Fluor 488 (dilution 1 : 300, A10667, Invitrogen Life Technologies) was added for 45 min at RT. Subsequently, after same washing procedure, the samples were incubated with phalloidin conjugated with ATTO-633 (dilution 1 : 50 in 1 mL PBS, Sigma Aldrich) for 1 hour at RT. Cell nuclei were counterstained with Hoechst 34580 (1 : 5000, H21486, Life Technologies) for 30 min at RT and washing twice in PBS. The samples were scanned using confocal microscope ZEISS LSM 5 DUO at wavelengths described above, and *λ*_ex_ = 633 nm, *λ*_em_ >650 nm for ATTO-633 staining.

### Real time PCR

RNA was isolated from four samples per group in day 0, 7, 14 and 21 using the RNeasy Mini Kit (Qiagen, Hilden, Germany) followed the manufacturer's instructor. RNA content (ng per sample) and ratio of absorbance at 260 nm to 280 nm, indicating RNA purity, was measured using Tecan Infinite® M200 Pro reader. Presence of the RNA in the samples was checked using standard agarose gel electrophoresis. The isolated RNA was processed followed manufacturers instruction. Briefly, osteogenic markers osteocalcin (OCN), collagen type I (COL1A2), integrin binding sialoprotein (ISBP), and EE1 (eukaryotic elongation factor, was used as housekeeping gene) genes were analysed. Detail description of the analysed genes in ESI Table S1.† For the reactions RevertAid H Minus First Strand cDNA Synthesis Kit (Thermo Scientific, Waltham, MA), TaqMan probes with a fluorescent label at the 5′ end and the quencher on the 3′ end (reviewed in ESI†), TaqMan Gene Expression Master Mix (Thermo Scientific, Waltham, MA, USA) and RT-PCR Grade Water (Thermo Scientific, Waltham, MA) was used. Results were generated from the values obtained by calculation according to the formula 2^−Δ*C*_p_^. Light Cycler 480 (Roche, Basel, Switzerland) was used to measure the fluorescence intensity.

### Statistical analysis

The data are presented as mean ± standard deviation. The results from the biological experiments were evaluated statistically using SigmaStat software (Systat Software, San Jose, CA, USA) by normality test and one-way analysis of variance (ANOVA). The Student–Newman–Keuls, Tukey, or Dunns method was used as *post hoc* test. Fluorescence intensity of collagen type I was measured from at least 8 confocal images. The intensity was normalised for glass and statistically evaluated using Multifactorial ANOVA where the level of significance was set as 0.05.

## Results

### Preparation of nanostructured surface

**Fig. 1:**
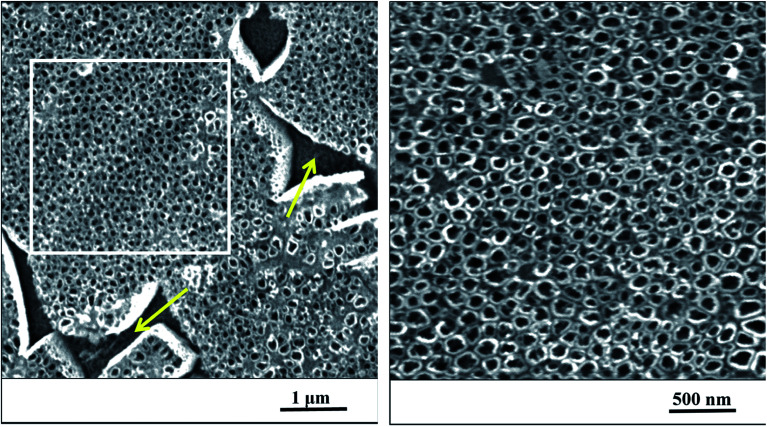
The nanostructured surface of Ti_6_Al_4_V alloy with cavities after beta phases (yellow arrows), SEM. Right image corresponds to the magnified marked square on the left.

**Fig. 2:**
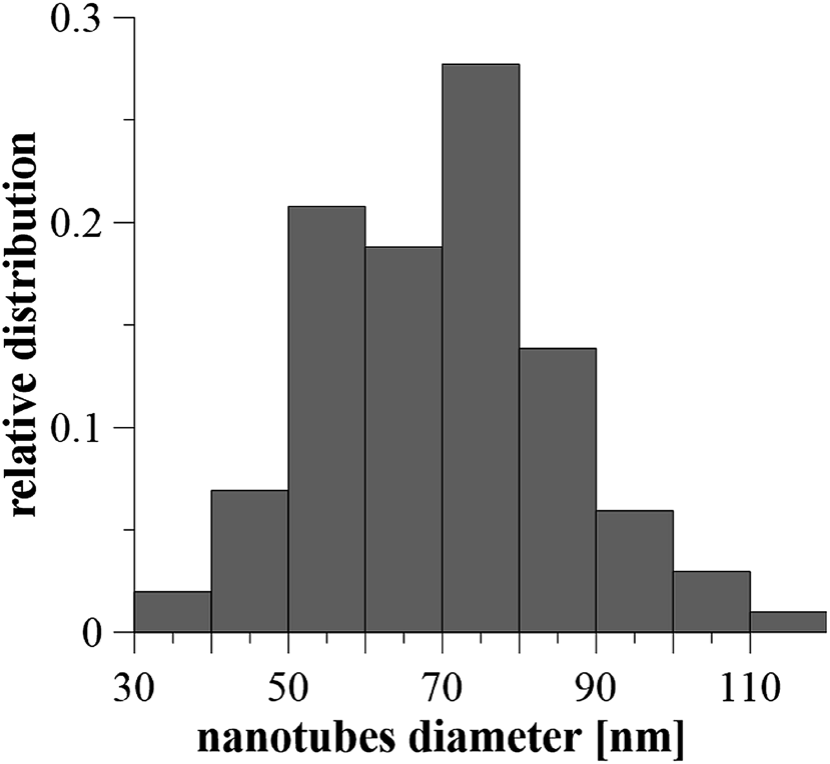
The relative distribution of nanotube diameters created on Ti_6_Al_4_V by anodic oxidation at 30 V. Diameters were evaluated from 100 areas on 5 samples.

After anodization in fluorides contained electrolyte, the nanostructured surface of Ti_6_Al_4_V alloy was obtained. Due to the presence of both α and β phases in the studied alloy, a non-uniform etching occurred, which led to the formation of cavities (arrows) after β phases (Fig. 1). The inner diameter of nanotubes ranged from 30 to 120 nm, the size between 50 and 80 nm was the most frequent (Fig. 2).

### Surface treatment

**Fig. 3:**
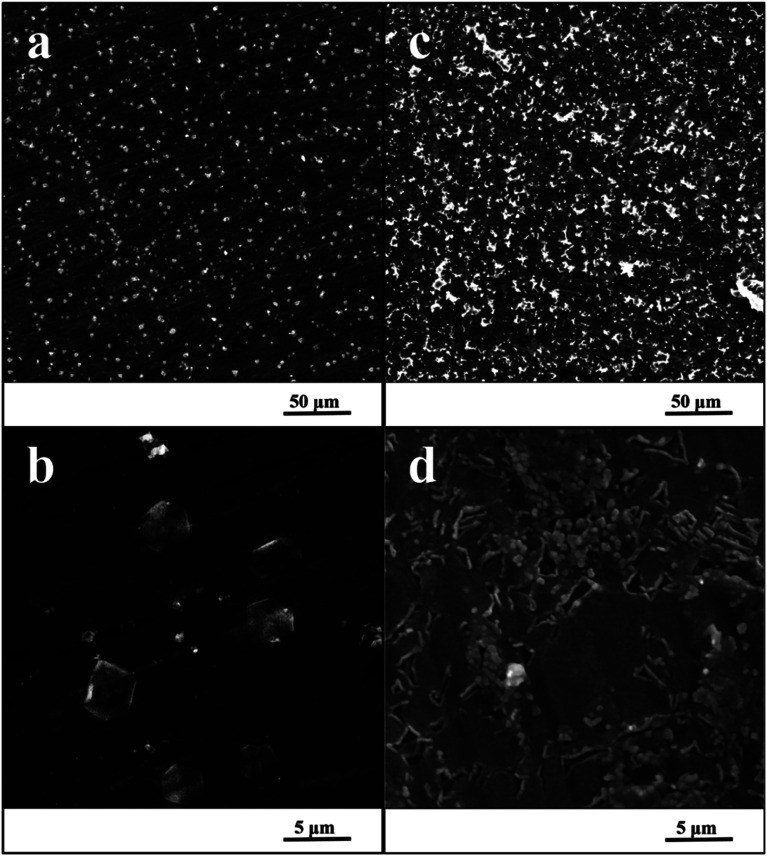
SEM image of ground specimen (a and b) and nanostructured specimen (c and d) after the exposure in SBF.

Fig. 3 shows the surfaces of specimens after the exposure in simulated body fluid. The EDS analyses detected the content of calcium and phosphorus in the precipitated layer on specimens, which was 1.5% at. of Ca and 0.9% at. of P for the ground surface. In case of the nanostructured surface, the content of these elements was higher, 9.7% at. of Ca and 6.1% at. of P. Moreover, EIS monitoring demonstrated that a faster hydroxyapatite precipitation occurs on the nanostructured surface (18 hours) compared to the ground sample (30 hours).

### Electrochemical measurements

**Fig. 4:**
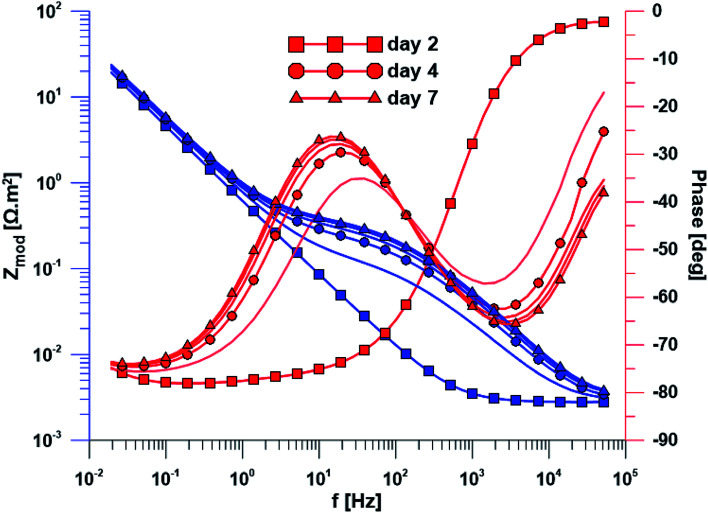
Electrochemical impedance spectra of the specimen with ground surface over exposure time.

The open circuit potential of the specimen with ground surface was stabilized at −220 mV/ACLE after approximately 30 hours of exposure. At the same time, the stabilization of polarization resistance also occurred. The induction period of he layer precipitation was also 30 hours, which was obvious from the impedance monitoring results at a constant frequency of 2 kHz (ESI Fig. S1†). The change in impedance spectra during exposure time is reflected in Fig. 4. The spectrum from the first day corresponds to the passive layer, the other spectra already show the change at the phase interface when the layer precipitated.

**Fig. 5:**
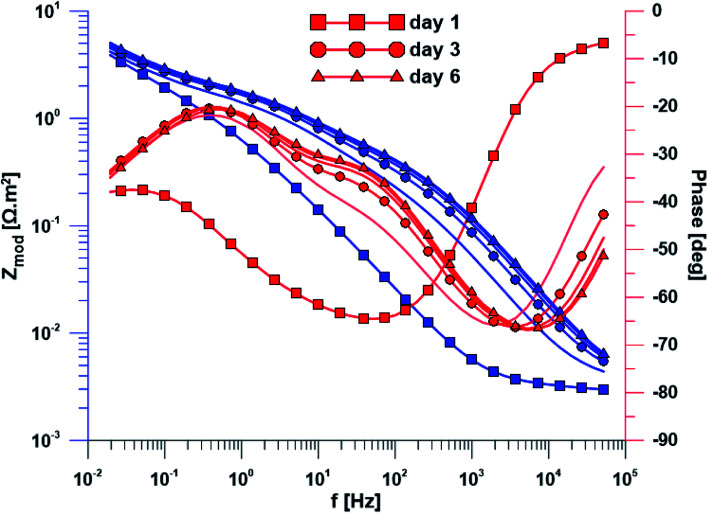
Electrochemical impedance spectra of the specimen with nanostructured surface over exposure time.

In the case of specimen with nanostructured surfaces, the time dependency of open circuit potential and polarization resistance was different in comparison with the ground surface. Open circuit potential was decreasing to −10 mV after approximately 50 hours of exposure time. In contrast, the value of polarization resistance was increasing as in the case of ground sample (ESI Fig. S2†). Also, the impedance spectra of nanostructured surfaces (Fig. 5) are different as compared to the ground alloy. Their shape is influenced by the charge transfer through the nanostructured surface. The change in surface condition occurred after 18 hours, what was evident from impedance monitoring.

### Cell response to Ti_6_Al_4_V surface modifications

**Fig. 6:**
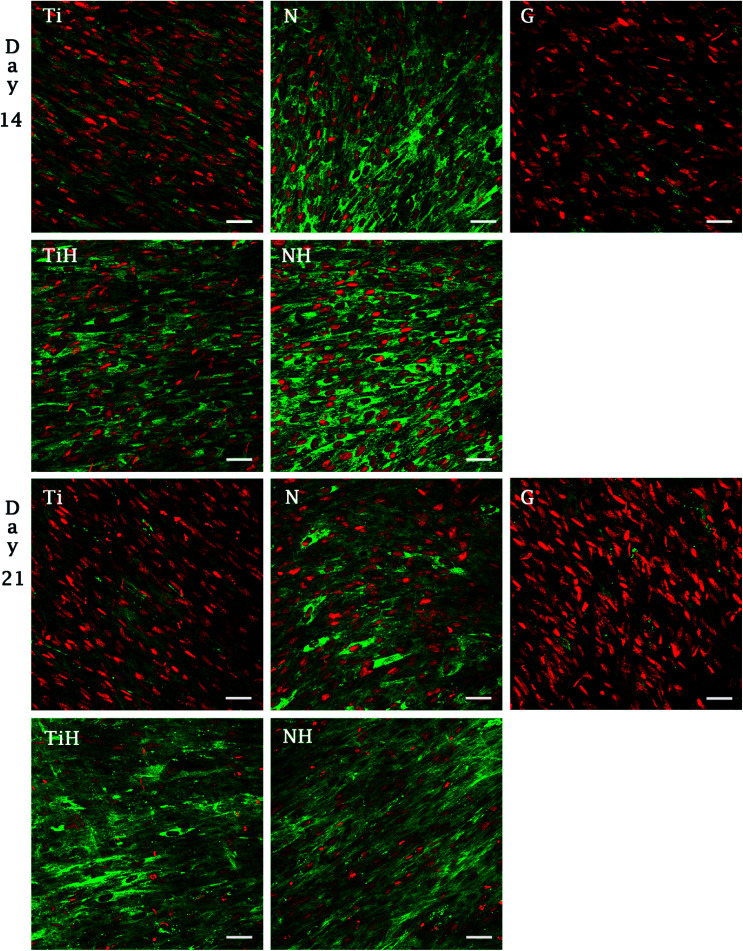
Visualisation of collagen type I synthetized by hMSC cultured for 14 or 21 days on Ti_6_Al_4_V samples with four different surface modifications: ground (Ti), nanostructured by anodic oxidation at 30 V (N), Ti_6_Al_4_V incubated in SBF (TiH), 30 V incubated in SBF (NH) and control glass (G). Immunohistochemical staining of type I collagen (green, Alexa Fluor 488) and cell nuclei (red, propidium iodide) was used. Confocal microscopy, objective ×20, magnification 1×, scale bar 50 μm.

**Fig. 7:**
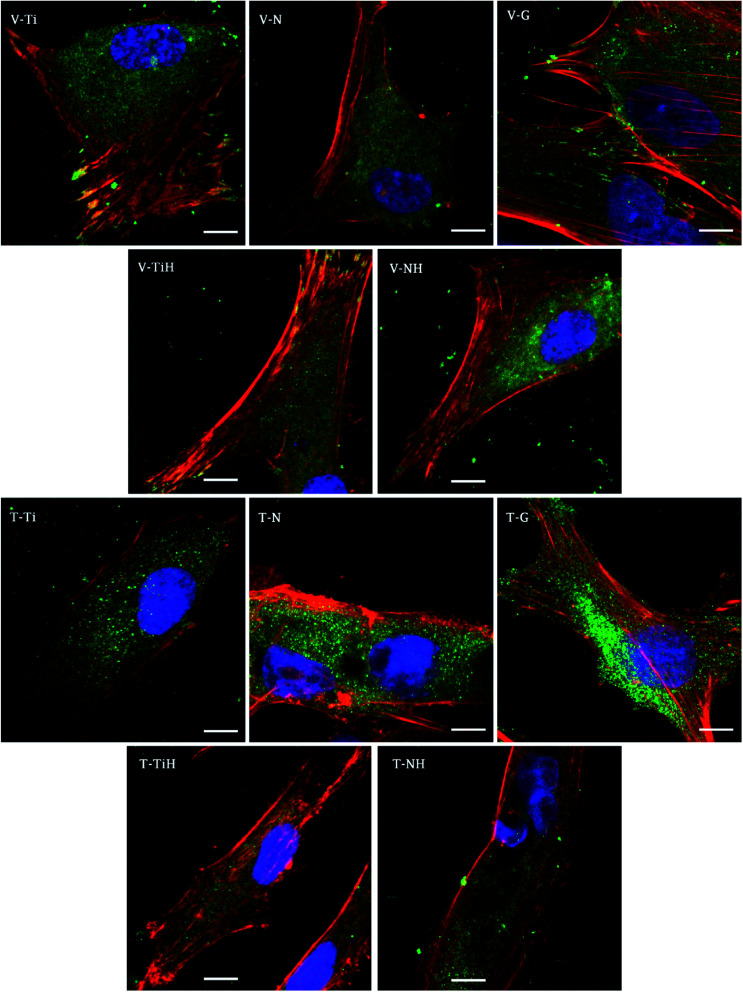
Visualization of adhesive molecules vinculin (V-) and talin (T-) in hMSC adhered on ground and modified Ti_6_Al_4_V scaffolds 48 hours after seeding. Cells cultured on ground Ti_6_Al_4_V (Ti), nanostructured Ti_6_Al_4_V by anodic oxidation at 30 V (N), Ti_6_Al_4_V incubated in SBF (TiH), nanostructured at 30 V and incubated in SBF (NH) and control glass (G). Confocal microscopy, objective 63×, magnification 2×, scale bar 10 μm, immerse oil. Staining: vinculin and talin (green, primary antibody and Alexa Fluor 488), actin cytoskeleton (red, phalloidin conjugated with ATTO-633), cell nuclei (blue, Hoechst 34580).

**Fig. 8:**
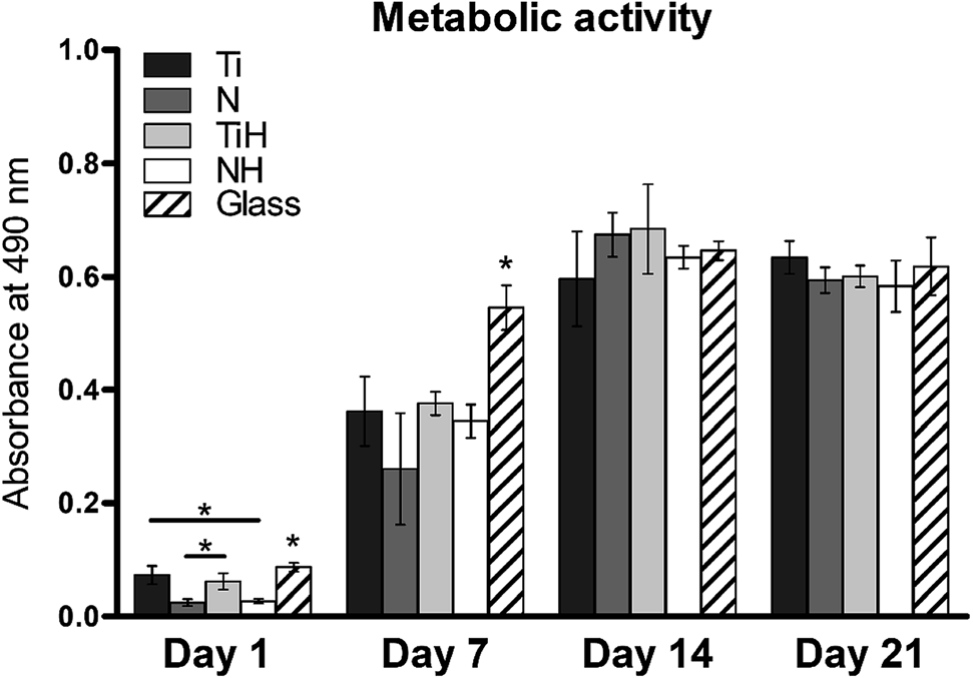
Metabolic activity of hMSC cultured on Ti_6_Al_4_V samples with four different surface modifications: ground (Ti), nanostructured by anodic oxidation at 30 V (N), Ti_6_Al_4_V incubated in SBF (TiH), nanostructured at 30 V and incubated in SBF (NH) and control glass coverslip. Statistical difference with *P* < 0.05 marked as *.

In order to investigate cell adhesion, growth, and differentiation on the specimens, hMSC from fourth passage were cultured on the specimens in osteogenic media for 3 weeks (Fig. 6). On day 2, well spread cells with polygonal morphology and developed cytoskeleton were seen on the specimens. As shown, microphotographs with immunohistochemical staining, diffused dot-like distribution of cytoplasmic vinculin and talin were detected in all samples. Matured focal adhesions were visible on Ti and TiH group, and on glass in distant parts of the cell membranes 48 hours after cell seeding (Fig. 7 and 8).

In this study, metabolic activity assay was employed to evaluate the viability of hMSC cultured on Ti_6_Al_4_V with four types of surfaces. 0 summarizes the absorbance of produced formazan by hMSC during 21 days of the culture. HMSC grown on all types of substrates, but significantly higher absorbance was measured on Ti and TiH when compared to both nanostructured samples N and NH on day 1. The highest values of metabolic activity were measured on glass on day 1 and 7. However, no significant differences in viability were observed among titanium samples on days 7–21.

**Fig. 9:**
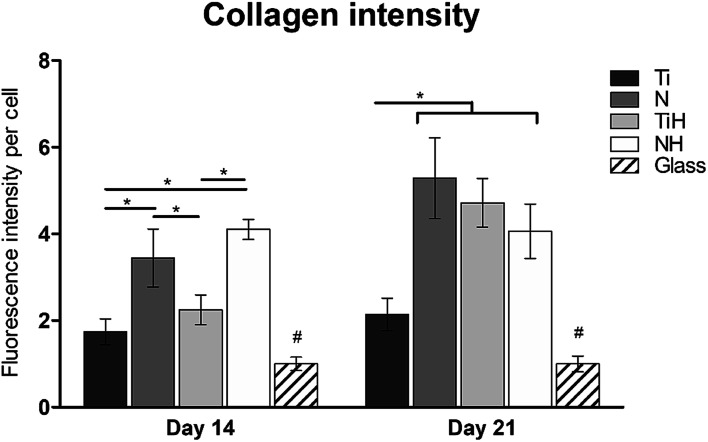
Collagen type I fluorescence intensity per cell in hMSC cultured on Ti_6_Al_4_V samples: ground (Ti), nanostructured by anodic oxidation at 30 V (N), Ti_6_Al_4_V incubated in SBF (TiH), 30 V incubated in SBF (NH) and control glass coverslip. Data were normalized against glass, statistical difference *P* < 0.05 marked with *, # significantly differs to all other groups.

The synthesis of collagen type I was evaluated from photomicrographs. As shows Fig. 9, nanostructures and HAp coating positively influenced collagen type I synthesis. On day 14, significantly higher fluorescence showed N and NH group compared to Ti and TiH. On day 21, higher collagen intensity was visible on both nanostructured samples and TiH than on Ti and glass. No significant difference was observed between N and NH on day 21.

The expression of osteogenesis-related genes and collagen synthesis were determined to assess the effect of surface modification on hMSC osteogenic differentiation. In parallel, 4 and 15 describe hMSC osteogenic differentiation trend cultured on four types of tested titanium surfaces and control glass coverslip for 21 days. It can be seen, that hMSC expressed *COL1A2* similarly on all tested surfaces; the significant differences were visible only on day 14 in case of Ti and glass. Additionally, up-regulated expression of collagen at late stage of differentiation (day 21) were on glass compared to all titanium samples. However, partial detachment of cells from glass during medium exchange was observed and growing cells might have influenced this effect. No significant differences were observed in mRNA expression of late osteogenic markers osteocalcin (*OCN*) and integrin-binding sialoprotein (*IBSP*). The high increase of *OCN* and *IBSP* expressions were measured in all groups. On day 0, almost zero expression of *IBPS* was found (not shown) (Fig. 11).

**Fig. 10:**
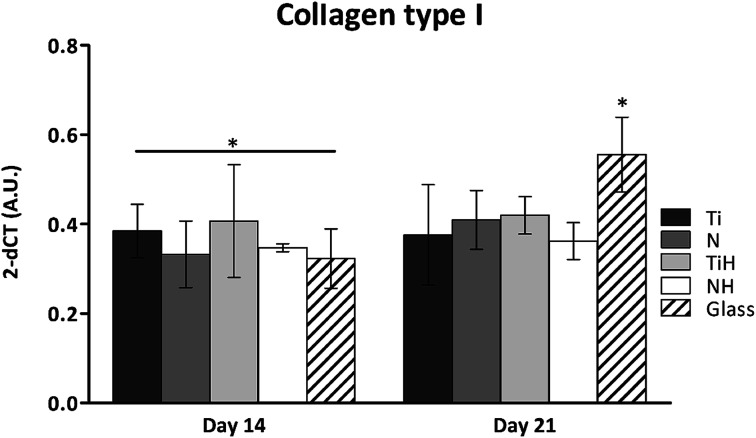
Collagen type I mRNA expression in hMSC cultured on Ti_6_Al_4_V samples: ground (Ti), nanostructured by anodic oxidation at 30 V (N), Ti_6_Al_4_V incubated in SBF (TiH), 30 V incubated in SBF (NH) and control glass coverslip. Statistical significance *P* < 0.05 marked with *.

**Fig. 11:**
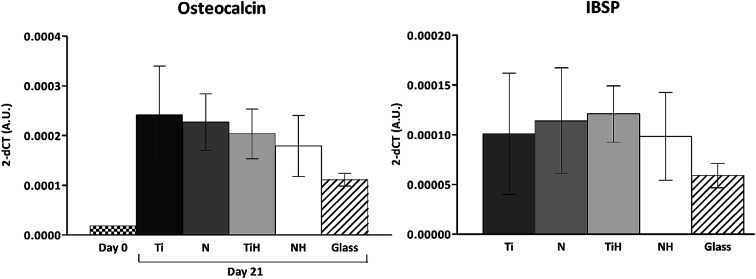
Expression of osteogenic markers in hMSC cultured on Ti_6_Al_4_V samples with four different surface modifications: ground (Ti), nanostructured by anodic oxidation at 30 V (N), Ti_6_Al_4_V incubated in SBF (TiH), 30 V incubated in SBF (NH) and control glass on day 21. On day 0 mRNA were isolated from cultivation plastic, IBSP – integrin binding sialoprotein. No significant differences on day 21.

**Fig. 12:**
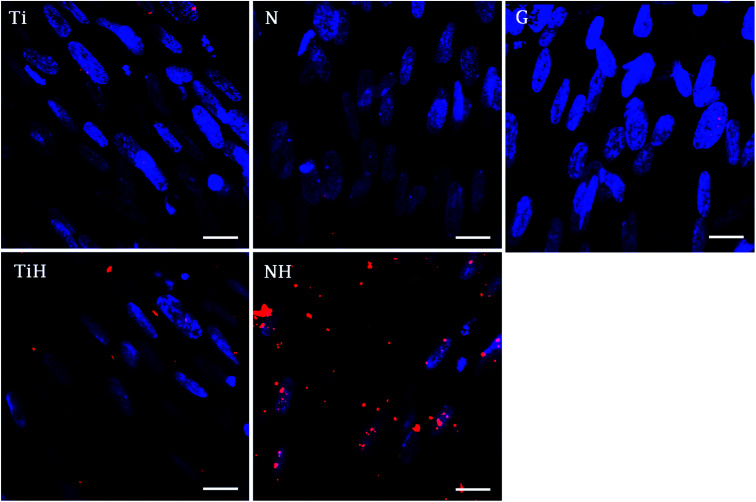
Photomicrographs of osteocalcin in hMSC cultured for 21 days on Ti_6_Al_4_V samples with four different surface modifications: ground (Ti), nanostructured by anodic oxidation at 30 V (N), Ti_6_Al_4_V incubated in SBF (TiH), 30 V incubated in SBF (NH) and control glass (G). Confocal microscopy, objective ×63, magnification 1×, scale bar 20 μm.

The immunohistochemical staining of osteocalcin, a late marker of osteogenic differentiation, showed that the highest density of osteocalcin was present on nanostructured samples exposed to SBF and lower amount on ground samples exposed to SBF. Other samples showed only faint osteocalcin staining (Fig. 12).

## Discussion

Our aim was to study the effects of hydroxyapatite layer, formed on different surfaces, on the adhesion, growth and differentiation of the human MSCs. Both nanostructure formation and hydroxyapatite layer formation have already been separately studied, yielding positive effects on both cell differentiation and bone formation.

In the first part of this study, the interaction of Ti_6_Al_4_V with simulated body fluid was investigated, when the two surface treatments were compared – ground and nanostructured surfaces. Many surface treatments of titanium alloys in order to enhance bioactivity and thus the cell differentiation and proliferation have been studied. Electrochemical oxidation under fixed experimental conditions in an electrolyte containing fluorides allowed the preparation of a well-defined and structurally arranged nanomorphology. Based on the previous study by Filova *et al.*,^15^ the oxidation process was managed at the potential of 30 V. In that investigation it was observed that nanotubes with the inner diameter of 50–80 nm obtained at the potential of 30 V had the best influence on bone cells adhesion, proliferation and differentiation. Also Oh *et al.* reported promoted MSC differentiation on nanotubes with diameter 70–100 nm thanks to stem cell elongation leading to cytoskeletal stress and enhanced differentiation into osteoblasts.^31^ Similarly, Lv *et al.* found that 70 nm was the optimal nanotube dimension for osteogenic differentiation of human adipose derived MSC *in vitro* and *in vivo*.

As mentioned above, Ti_6_Al_4_V is consists of α and β phases. The β phase is less corrosion stable than α phase because it is enriched with highly soluble vanadium compounds, thus β phase is etched preferentially. The area of the cavities covers approximately 11% of the whole surface as mentioned by Moravec *et al.*^32^

A wide range of electrochemical methods can be used to study the interaction of biomaterial with the human body environment. Open circuit potential for both ground and nanostructured specimens decreased until its stabilization at the value of −220 mV/ACLE in the case of ground surface and later at the value of −100 mV/ACLE for the nanostructured surface. Increasing polarization resistance values for both surface treatments could be explained by phase interface stabilization. The lower resistance value of the nanostructured surface is caused by the much larger surface of the nanotubes as compared to the ground surface.

Another method used was electrochemical impedance spectroscopy, which provides accurate and high-quality information on phase interfaces. The different shape of the impedance spectra is obvious when comparing the ground and nanostructured specimens. In the case of the ground sample, the spectrum from the first day corresponds to the passive layer and the other spectra already showed the change at the phase interface when the layer precipitated. The spectra obtained while exposing the nanostructured specimen have a totally different character; their shape is influenced by the charge transfer through the nanostructured surface.

Based on the measurement of impedance spectra over the entire frequency range, a 2 kHz frequency value was selected at which the greatest variances in phase angle and impedance module were detected. From the measurement of these parameters at a constant frequency of 2 kHz, it was possible to accurately evaluate the time of change on phase interface, hydroxyapatite (HAp) layer formation and growth was anticipated. This assumption was confirmed by EDS analyses after the exposure to SBF, when Ca and P were detected at an atomic ratio almost 1.67. The positive effect of the nanotubes is obvious from the single frequency results; the induction period of the nanostructured surface was about 12 hours shorter than that of the ground sample. Higher amount of Ca and P measured by EDS also confirmed the enhanced bioactivity of the nanostructured surface.

Also, the SEM microphotographs clearly demonstrated that formation of HAp-like layer was greatly enhanced by nanostructured surface. This outcome is in accordance with previous studies.^29,33^

From the biomedical point of view, the use of Ti_6_Al_4_V alloy for bone replacements could be occasionally viewed with apprehension, as elements Al and V are known for their possible cytotoxic effects.^34–37^ In earlier studies dealing with the form of these two elements in the nanotubes^15,32^ it was confirmed that Al is present in the form of Al_2_O_3_, also known as alumina, which is a widely used biomaterial that positively affects the adhesion and growth of osteoblasts,^38^ especially in the form of nanoparticles. In the case of V, this element was detected as oxide V_2_O_5_. Since the effects of V_2_O_5_ are more controversial.^39,40^

As a hydroxyapatite layer is more natural for cells than artificial surfaces, the surface covered by it may have improved biocompatibility and bioactivity. 3D-HAp/poly-d/l-lactide composites showed earlier osteoblast differentiation compared with β-TCP.^41^ In collagen composites with HAp, the osteogenic differentiation was influenced by concentrations of collagen, HAp, and pore diameter, with the best results on composite with 50 wt% Hap and 0.05% collagen.^42^ Similarly, Sun *et al.* reported better osteogenic differentiation of rat BMCs on collagen/HAp (60 wt% HAp) scaffolds than on β-tricalcium phosphate/Hap or pure HAp scaffold.^43^

Previous studies shown that micro- and nano-patterned HAp bioceramic surfaces enhance cell attachment and can stimulate osteogenic differentiation or bone regeneration.^44,45^ As an example, the micropatterned HAp surface showed strong osteoblasts contact guidance effect on 8 μm width microgrooves, whereas on larger patterns the cell alignment in the grooves direction proved to be weak.^46^ In another study, microgrooved HAp ceramic was applied to direct growth of osteoblasts.^47^

It was also shown that micro and nanostructures on bioceramic activate expression of different stem cell integrins. Zhao *et al.* described the synergic effect of hybrid micro/nanostructured surface of HAp bioceramics on osteogenesic differentiation of hBMSCs (human bone marrow stem cells). In addition, their results also revealed significantly enhanced cell adhesion, proliferation and expression of osteogenic genes on both patterned surfaces, compared to flat control.^48^

HAp coating with nanorod-structures on Ti_6_Al_4_V implants were applied to promote cell adhesion and osteointegration in diabetes mellitus rabbit models.^49^ The importance of distinct architecture of the biomaterial surface were recently reviewed. Cell fate is guided by the micro and nano patterned substrates, which influences protein adsorption, cell adhesion, migration, growth, differentiation and cell death. Various cell types differently react on the micro or nano patterned surfaces.^50^ In the study of Özçelik *et al.* BMSCs preferred smooth surface while osteosarcoma Saos-2 cells preferred nanopillar surfaces. However, the presence of fibronectin improved the BMSCs adhesion on nanopillar surfaces.^51^ The different behaviour of osteoblasts and myoblasts were strongly influenced by the width of HAp microgrooves prepared by photolithography, and subsequent coating with HAp.^46^

It is believed that nanotubes offer greater surface area for cell-biomaterial contact, and therefore better mimic bone architecture and function.^13,14^ Another approach to promote bone regeneration and new bone formation from the surrounding native bone is surface coating using SBF that create calcium phosphate structures similar to HAp of native bone.^52,53^ It was supposed that combining these two above mentioned surface modifications could greatly influence osteoinductivity and osteointegration of the Ti_6_Al_4_V implants.

For orthopaedic and dental implants success, not only osteoblast cells but also MSC play a key role in bone tissue regeneration. Recently, multipotent cells are becoming preferred model in a tissue engineering studies and hMSC are widely used as an osteogenesis model and for bone-tissue engineering applications. Therefore, in the current study hMSC were chosen to investigate the bioactivity of the created surfaces *in vitro*. NanoHAp coating of Ti specimen supported growth and osteogenic differentiation of hBMSC, which was enhanced by the addition of growth factors.^54^

From the previous studies it can be concluded that by specifically designing the nanotube diameter it is possible to support either hMSC adhesion or osteogenic differentiation.^55,56^ As was shown larger nanotube diameters lead to elongation of the cells and stimulate osteogenic differentiation *via* cytoskeletal stress.^31^ Moreover, it was clinically proven, that rough surfaces induce a more rapid osseointegration than the smooth surfaces^57–60^ and some of the porous Ti-based biomaterials are already in clinical trials.^61^

A crucial step for implant stability and subsequent cell differentiation is initial cell adhesion. This process is initiated by the adsorption of proteins from the body fluids to the implant surface that are recognised by the cell integrins, as the first step of the cascade leading to the formation of adhesion plaques. These highly organized protein complexes mediating ECM-cell contact but also act as mechanosensors that regulate cell phenotype.^62^ As was mentioned, focal adhesion complex consists of many proteins, where the mostly considered are talin, that forms a structural core of the focal adhesion, and vinculin which strengthens and stabilizes the focal adhesion complex.^63,64^

This phenomenon can be related to the fact that MSC were shown to adhere better on smaller nanotubes (30 nm), while larger nanotubes (100 nm) induced stem cell differentiation and proliferation.^65^ Although nanotubes with larger diameters were shown to be osteoinductive without the use of osteogenic supplements,^13^ other aspects such as cell sources, adsorbed proteins and their conformation, age and even the sex of the donor may influence cell response.^66,67^

In this study, cell attachment and morphology were evaluated 48 hours after seeding. Well spread polygonal-shaped cells were visible on all type of surfaces. Additionally, the distribution of molecular markers of cell adhesion, talin and vinculin, were evaluated by immunofluorescence staining. As shown in Fig. 7, vinculin was relatively diffused with well-developed focal adhesions on Ti and TiH group. Cytoplasmic talin was captured, but focal adhesions were not clearly visible.

Distribution of the vinculin positive areas in our work agreed with the study conducted by Saha *et al.* who showed dot-like cytoplasmic localisation of vinculin in osteoblasts on nanostructured Ti_6_Al_4_V^68^ and with our previous study using cpTi.^69^ We have shown similar dispersal of vinculin and talin in osteoblasts cultured one day on nanotubes with diameters among 24–66 nm.^69^

The study conducted by Filova presented the increase of both talin and vinculin concentrations in osteoblasts on nanotubes created by anodic oxidation at 30 V with an inside diameter around 35–80 nm compared to untreated Ti_6_Al_4_V.^15^ Costa *et al.* found that as roughness and complexity of HAp coated PCL matrices increased, the number of focal adhesions decreased.^70^

Higher vinculin staining implies that more focal adhesions were present in hMSC cultured on treated surfaces. The data suggest that increase in roughness could lead to enhanced initial adhesion. Similarly, increased formation of focal adhesions due to nano-roughened surface has been reported in osteogenic cells.^71^ We suggest that less developed focal adhesions on NH group could be caused by the phenomenon that nanotubes were overlapped by the HAp-like layer created by SBF treatment, therefore the simultaneous effect of nanotopography and HAp crystals could not be clearly detected. Our data indicating that talin synthesis or morphology were influenced by presence of the HAp. Talin intensity was lower on TiH and NH samples (Fig. 7) Colocalised F-actin and vinculin signal was visible only on Ti and TiH and glass group, but not on nanostructured groups which corresponds to our results on cpTi.^69^

A possible explanation of different results obtained from previous studies reporting focal adhesion formation,^53,71,72^ could be the shorter time of cultivation and possibly different type of integrins expressed in hMSC and other osteoblast cells.^66^ It has been shown that various types of integrins are expressed in osteoblasts.^73^ The type of expressed integrins subsequently determine important downregulated signalling pathways resulting in different cell fate and behaviour.^62^ Surface dependent expression of integrins described Brugge *et al.* in rat bone marrow cells cultured on titanium implants with various surface treatments.^74^ Other studies, considering substrate-dependent integrin expression, have shown that surface energy, chemistry and structure can fundamentally influence osteoblasts fate on titanium implants throughout integrin expression.^75–78^

In the present study, MTS assay was employed to evaluate the metabolic activity of hMSC cultured on different substrates. The method is based on the reduction of yellow MTS substrate by mitochondrial enzymes in living cells to water soluble purple formazan.

MSC grown on nanostructured surfaces NH and N displayed lower metabolic activity when compared to those cultured on Ti, TiH and glass on day 1. However, cell metabolic activity increased significantly in all samples and was similar on the titanium substrates from day 7 till day 21. It has been documented, that hMSC cultured on rough Ti surfaces decreased in cell number after 5 days of culture compared to smooth surfaces. The authors suggest that lack of proliferation was caused by the selection of hMSC compatible with growth on rough Ti surface or committed to osteogenic lineage, from phenotypically heterogenous hMSC population.^79^ Considering this, the observations of decreased cell numbers on TiH and N group are therefore no surprising.

To investigate the influence of modified titanium substrates on hMSC differentiation, osteoblast differentiation associated genes *COL1A2*, *IBSP*, and *OCN* were evaluated together with detection of selected proteins by immunohistochemical staining. Upregulated collagen type I synthesis was evident on both N and NH surfaces on day 14 and on all modified samples on day 21 (Fig. 10). Comparison of ground Ti and treated samples did not show any significant up-regulated expression of selected genes towards modified surfaces. However, the highest amount of mRNA for *IBSP* and collagen type I was found for TiH group, with the same trend in *COL1A2* expression on day 14. High values of *IBSP* indicate the presence of terminal osteoblastic differentiation. *IBSP*, a non-collagenous osteogenic matrix protein, also known as marker of both terminal osteoblastic differentiation and new bone formation, is also believed to be involved in hydroxyapatite nucleation.^80^

On day 14, the exposition of Ti and N samples to SBF did not increase the collagen type I intensity on TiH and NH samples. Moreover, nanostructured samples showed significantly increased collagen intensity than other samples. However, on day 21, both nanostructured and HAp-modified samples showed a significant increase of collagen intensity compared to unmodified Ti and glass. Thus, the collagen formation is more influenced by nanostructured surface than HAp layer formation.

In our previous study, higher collagen synthesis on larger nanotubes (66 nm) compared to both smaller diameters and untreated surface were described.^69^ Current data correlate with upregulated collagen synthesis and no change in osteocalcin expression described previously on nanostructured implant surface *in vivo*, indicating an accelerated bone formation around the nanotubes.^81^

Furthermore, osteocalcin as an extracellular protein produced by differentiated osteoblast, indicating late stage of differentiation and mineralization stage.^82^ Although osteocalcin expression was not significantly affected by hereby used modifications, the numerous signals was visible on NH and less rare on TiH samples, compared to very rare signal on Ti but no signal on N and glass on day 21. The presence of osteocalcin (positive red signal in Fig. 12) suggests that MSC had differentiated into mature osteoblasts preferentially, mainly on HAp-like coated surfaces.

Oh *et al.* reported osteoblastic differentiation of human MSCs on nanotubes with higher diameter (70–100 nm) of nanotubes, but no osteogenic differentiation on nanotube with small diameter about 30 nm. Conversely, Park *et al.* reported better osteogenic differentiation of hMSC on 15 nm-diameter TiO_2_ nanotubes.^83^

The study conducted by Habibovic, showed positive effect of calcium phosphate coated Ti_6_Al_4_V on bone healing *in vivo*. Authors suggested that presence of calcium phosphate is important for ectopic bone formation.^84^ Also Barrere *et al.* reported enhanced bone integration in case of SBF mediated apatite-coated Ti_6_Al_4_V compared to noncoated implants on goat models.^85^ Cai *et al.* reported titanium (cpTi) treatment with potassium hydroxide and heat, and subsequent exposure to SBF and its effects on the rat BMSCs. The samples treated with potassium hydroxide, heat and exposed to SBF showed significantly higher metabolic activity, alkaline phosphatase activity and osteocalcin synthesis compared to pure cpTi. Samples treated with potassium and heat but not exposed to SBF did not show positive effect on BMSCs osteogenic differentiation.^86^

Considering above mentioned studies and our data, the SBF mediated mineralised surfaces of metal implants and other biomaterials have great future application, as this approach can be used for drug delivery that can dramatically enhance tissue regeneration. For instance, implant surfaces can be functionalised by the incorporation of bioactive molecules, such as antibiotics, growth factor and proteins, even nucleotide acids that can be used in gene therapy.^52^

In this study, we showed that nanostructured Ti_6_Al_4_V exposed to SBF was able to stimulate collagen type I and osteocalcin synthesis in hMSC, together with a high rate of cell growth.

## Conclusions

In summary, anodic oxidation at 30 V created nanotubes with inner diameters ranged from 30–120 nm on titanium alloy Ti_6_Al_4_V. Subsequently, SBF exposition was applied to form HAp-like layer on the ground and nanostructured titanium surface. In this study, SEM microphotographs clearly demonstrated that formation of HAp-like layer was greatly enhanced by nanostructured surfaces and covered almost the whole surface of the specimens. Our data showed that the hereby presented titanium surface modifications supported proliferation and osteogenic differentiation of hMSC. In particular, treatment of ground Ti_6_Al_4_V with SBF resulted in enhanced collagen synthesis, similar to nanotubes modified samples after a longer cell culture. In addition, nanotubes exposed to SBF enhanced osteocalcin synthesis better than other surfaces. Our data suggest that Ti_6_Al_4_V nanostructuring together with SBF incubation can enhance extracellular matrix formation and thereby can improve bone regeneration.

## Conflicts of interest

There are no conflicts to declare.

## Supplementary Material

RA-010-C9RA08912H-s001
