# Supplementary material for: Mesenchymal stem cell interaction with Ti6Al4V alloy pre-exposed to simulated body fluid
Source: RSC Adv. 2020 Feb 13;10(12):6858–72. doi: 10.1039/c9ra08912h (PMC9049760; doi:10.1039/c9ra08912h)
Supplement: RA-010-C9RA08912H-s001 [file RA-010-C9RA08912H-s001.pdf]

Electronic Supplementary Material (ESI) for RSC Advances.
This journal is © The Royal Society of Chemistry 2020

Supplementary data

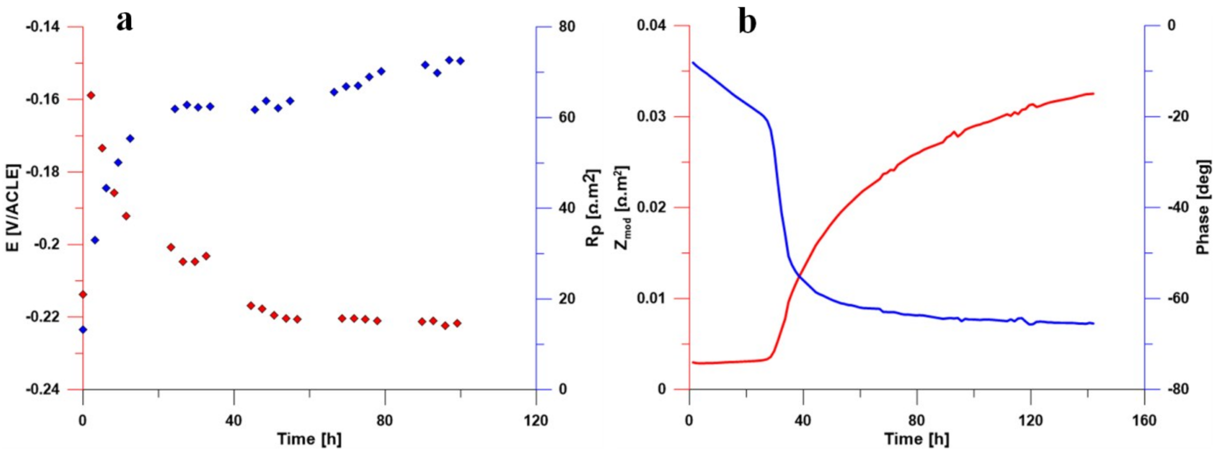

Figure S1. Time dependency of open circuit potential and polarization resistance (a) and electrochemical impedance spectroscopy at frequency 2 kHz for the specimen with ground surface (b).

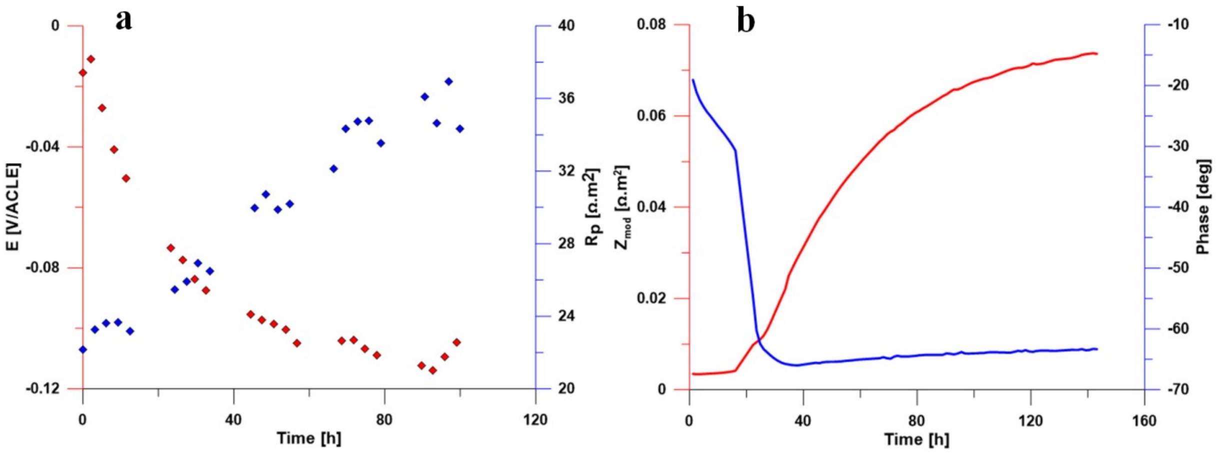

Figure S2. Time dependency of open circuit potential and polarization resistance (a) and electrochemical impedance spectroscopy at frequency 2 kHz for the specimen with nanostructured surface (b).

Table S1. List of analysed genes with their codes and catalogue numbers.

Gene name	Code	Catalogue Nr.
EEF-1	Hs00265885_g1	4331182
COL1A2	Hs01028969_m1	4331182
IBSP	Hs00913377_m1	4331182
OCN	Hs01587814_g1	4331182
RUNX2	Hs00231692_m1	4331182

*COL1A2-collagen type I alpha 2 chain, EEF-1- eukaryotic translation elongation factor 1 alpha 1, IBSP- integrin binding sialoprotein, OCN-osteocalcin, RUNX2-runt related transcription factor 2
